# Patient and public involvement in rheumatic and musculoskeletal research: an idea whose time has firmly come

**DOI:** 10.1186/s41927-023-00340-z

**Published:** 2023-05-31

**Authors:** Angie Botto-van Bemden, Adewale O. Adebajo, Ciarán Martin Fitzpatrick

**Affiliations:** 1 Global Patient Ambassador, Musculoskeletal Research International, Inc., Miami, FL USA; 2 Patient Partner, Holiday, FL USA; 3 EUPATI Fellow, Holiday, FL USA; 4 Clinical Research Experts, LLC., Tampa, FL USA; 5 Faculty of Medicine, Dentistry and Health, University of Sheffield, Sheffield, UK; 6 BMC Medicine, The Campus, 4 Crinan Street, London, N1 9XW UK

## Abstract

Patient and public involvement is an idea whose time has firmly come. It is the views of these Guest Editors that it is the right thing to do morally and improves research quality and applicability.

## Background

How can young investigators/clinicians ensure their research is equitable, meaningful and improves healthcare for all, which translates bench evidence to bedside action/guidelines/policies/reimbursement and beyond? PPI elicits the voice of the patient and shares their lived experiences. It influences their individual value attributes—priorities, needs, expectations and preferences of patients that are all formed from their experiences. PPI demonstrates collaborative research between researchers and patients and/or members of the public rather than to, about, or for them (National Institute for Health Research, UK). Patients and the public may be partners on the research team or leading research themselves (i.e. user-led research). PPI is no longer a novel concept but a shifting paradigm credited to increasing awareness and requirements placed on researchers by patients, scientific societies, funding agencies, and, more recently, regulatory and health technology assessors. Notably, the dynamic, heterogeneous and multifaceted nature of lived experiences encourages researchers to partner with patients early and during research if their true North is to learn from patients and effectively improve health for all.

Musculoskeletal and rheumatic healthcare practitioners have played a leading role in developing strategies to engage patients as authentic partners in clinical practice and research; however, many basic researchers have expressed needing knowledge and resources to contact, plan or manage meaningful PPI. Last year, *BMC Rheumatology* and *BMC Musculoskeletal Disorders* announced a Call for Papers on 'Patient and public involvement (PPI) in rheumatic and musculoskeletal research' to provide a constructive learning experience and involve public partners more effectively. All papers related to PPI were welcomed and considered.

The 31 articles published in this Article collection can be categorized into several PPI-related themes spanning all stages of research—patient education/empowerment/engagement/perspectives, research priority setting, co-created design, improving outcomes, patient-led design, harnessing clinical care frameworks, community/patient advisory boards and quality evaluation. First, Arumugam et al. [1] provided a brief review introducing the background of PPI along with practical considerations, referencing frameworks, guidance and tools for researchers to get started—most notably, a helpful table was created with links to resources as an additional file for help integrating PPI throughout the research lifecycle.

Integrating PPI in research offers an ideal opportunity to promote health equity. Patient education and capacity building are the first steps researchers can take to improve health literacy and numeracy. Marinello et al. [2] incorporated PPI into their practice via a new BehçeTalk Programme, empowering rare disease patients through education and capacity building to promote active involvement in therapeutic decision-making. Singhal et al. and Elliott et al. focused on engaging young people in research to improve patient-reported outcome measures and communication [3, 4]. Esen et al. [5] went one step further, sharing the voices of young people in rheumatology research via the development of an enduring national youth advisory group that is active and eager to co-create throughout all stages of the research cycle.

In addition to patient engagement and education, their expectations and preferences influence adherence to treatment programs. Myers et al. [6] described a protocol using the Patient Engagement, Education, and Restructuring of Cognitions (PEERC) intervention intended to change expectations regarding conservative care where surgical and non-surgical interventions have similar results. Akesson et al. [7] shared measurements of enablement and empowerment amongst participants in their supported osteoarthritis self-management program, suggesting others may also want to consider incorporating such tools as measurable outcomes. Dorris et al. [8] demonstrated the value of PPI in discovering previously unrecognized areas of research during research priority setting with all stakeholders in rheumatic and musculoskeletal diseases (RMD), noting novel priorities of mental health, pain and diet. Other contributors shared insights from specific RMD cohorts. Vitaloni et al. [9] shared findings from the perspectives of patients with osteoarthritis, prioritizing the need for better biomarkers, earlier diagnosis, management monitoring, and non-surgical management options. Williams et al. [10] shared formative research findings to promote lupus awareness and early screening at Historically Black College and University communities.

Many authors shared research integrating PPI to improve outcomes and adherence. Teo et al. [11] shared factors affecting patient engagement in exercise rehabilitation, noting that exercise programs are best co-designed between patients and exercise specialists with individual consideration of engagement factors to optimize participation and outcomes. Middlebrook et al. [12] delved further into improving musculoskeletal trauma recovery outcomes by evaluating patients' and physiotherapists' successful recovery perceptions to ensure alignment. Salmasi et al. [13] reported on researchers' perspectives in an OMERACT adherence study, while Cornelissen et al. [14] shared a co-designed multi-component adherence protocol for osteoporosis care.

Patients were not solely research partners; patients also drove and led PPI. Jethwa et al. reported on a patient-driven pilot survey which collected patient perspectives on telemedicine appointments during the COVID-19 pandemic. Currie et al. [15] shared a patient-led qualitative study in juvenile idiopathic arthritis to help transition from juvenile to adult rheumatology care [16]. Kee et al. and Oyebanjo et al. both highlighted PPI in clinical care frameworks. Kee et al. [17] engaged patients to participate in defining best-practice rheumatology service provision in New Zealand, while Oyebanjo et al. [18] shared navigation of a patient's journey for outpatient consultations in Britain to co-create a patient-led clinic visit framework. Bech et al. [19] continued the theme of improving care frameworks via a patient-initiated follow-up study as reorganized support for increased patient involvement.

Moving beyond single-study settings, Sagen et al. [20] noted that Norwegian policy states that patient participation is one of six dimensions defining healthcare service quality. Patient advisory boards (PABs) are a statutory part of rehabilitation institutions to meet these patient-centered demands. Noting the lack of general rules or procedures for PABs, Sagen et al. [21] conducted an exploratory study to learn how patient representatives experienced their organization, influence and impact. Findings confirmed that rehabilitation institutions improved with enduring PPI via PABs, further suggesting that rigorous evaluation must continue to ensure continued quality. Quirk et al. presented the assessment of their community-based initiative for engaging people with long-term health conditions through physical activity—the Parkrun PROVE project. This project utilized a PROVE project manager partnered with patient outreach ambassadors. The findings above are relevant for researchers wanting to cooperate with community-based organizations wishing to implement similar outreach initiatives. Recommendations, resource management, communication, leadership, volunteer autonomy and tips for defining and capturing success were included in both pieces.

Researchers whose goal is to ensure effective patient partnering throughout projects are encouraged to refer to the Rheuma Tolerance for a Cure (RTCure), a five-year, international and transnational collaboration [22], as well as de Wit et al. [23], which evaluated the added value of PPI, pitfalls and conditions for success in one basic rheumatology institute in The Netherlands. Both articles shared their comprehensive PPI-integration examples, reviewed and highlighted PPI opportunities for success, which may be applicable to basic and translational environments.

Ideally, PPI in research leads to co-created evidence-based guidance, clinical practice guidelines, and effective patient decision aids for use in shared-decision making. Implementation must also be co-designed with PPI to ensure equity and quality of life. Vitaloni, Botto-van Bemden et al. systematically reviewed evidence reporting individual factors affecting quality of life in patients with knee osteoarthritis, so healthcare professionals were delivered data on psychosocial factors that aid patient management strategies [24]. Babatunde et al. [25] scoping reviewed the implementation of PPI in evidence-based guidance for musculoskeletal conditions, noting significant variations in care, current advances and areas needing improvement. Lange et al. [26] disseminated their protocol integrating PPI into clinical practice for knee replacement utilizing an individualized decision aid. Their research addresses the absence of patient expectations in international guidelines and aims to provide that evidence. Patient expectations are modifiable and may improve decision quality when included into shared decision-making. Their tool may provide physicians with patient-specific and disease-specific factors along with treatment goals and preferences for indication, allowing for individualizing treatment options according to patient preferences and needs.

Finally, PPI integration in research must be continually evaluated and improved upon. The Public and Patient Engagement Evaluation Tool is one such measure. Garratt et al. [27] provided an example of translating and culturally adapting this tool in Norway. Importantly, patient-led research collaborations most recently published scorecards for patient engagement which researchers are encouraged to access [28]**.**

The Article collection ranged from priority setting to co-created protocol design with patients as research partners, including public authorship and reviewing. Most importantly, this collection goes beyond mere PPI insights and shares advances, including clinical guideline implementation using a participatory approach, measurement tools for patient enablement and empowerment, and engagement via community-based initiatives. The patient perspective was paramount throughout this process, offering considerations for improving PPI value, impact and gaps. All commissioned articles were co-produced with patients, and patients were invited to referee all peer-reviewed manuscripts. We salute this alliance and efforts to ensure robust and fair open science, who educated the Editorial team and encouraged authors to include plain-language summaries and the GRIPP-2 reporting checklist.

## Conclusions

This PPI in RMD selection shall particularly interest all stakeholders in rheumatology and musculoskeletal, although learnings and tips can be applied to other fields. The time for routinely integrating PPI into aspects of the healthcare cycle is now. Collectively, healthcare and research communities must advocate for the agenda and funding to sustainably integrate PPI into a unified and supportive infrastructure for equitable action!

## Data Availability

N/R.

## Abbreviations

PAB: Patient advisory boards
PPI: Patient and public involvement
RMD: Rheumatic and musculoskeletal disease
